# Oral Antisepsis

**Published:** 1888-07

**Authors:** John L. Gish

**Affiliations:** Jackson, Mich.


### Oral Antisepsis.

BY JOHN L. GISH, M.D., D.D.S., JACKSON, MICH.

[Read before the Michigan State Dental Association, at Ann Arbor, Mich., March 20, 1888.]

There is no one subject at issue in our dental profession to-day that should receive more thought and consideration than the subject of dental or oral antisepsis. Dental caries has been the subject of much careful thought and investigation for many years past. Our knowledge of dental caries has been greatly enlarged by the associate sciences of microscopy and chemistry. It was by the aid of these sciences that we learned that micro-organisms were always present in and around a carious tooth, and that it was acid; and yet we could not declare that these organisms were the true cause for they were present under all circumstances, and it was equally as fair to assume that the acidity might be the *effect* rather than the cause of the diseased state.

The chemist, long before the microscopist, proved the presence of micro-organisms, discovered that in the process of decay a class of substances, having distinct chemical and physiological properties were formed. Later, this class of bodies were found to be analogous with the so-called *alkaloids* and called ptomaines, but their mode of formation and their true chemical composition was not discovered.

It is still a matter of much consideration as to how the ptomaines are formed, some claiming that it is the excreta of the microbes; others that it is the result of the breaking up of more complex substances, or the union of simpler bodies as the result of the disturbance of the molecular state of the compound caused by the presence and growth of the micro-organisms. The almost indefinite number of forms and wide distribution of microscopic life are known to all. We find them in the earth, in the air, and in the water.

We know that certain species or families of microscopic life, produce during their development, bodies or substances which, according to our modern chemistry, are classed among acids; and acids are hydrogen compounds in which hydrogen has a weak affinity and is more easily liberated than the other elements of its group.

We know that all forms of life, however small or however great, are produced and reproduced either for better or for worse when existing under the proper circumstances and conditions. Therefore, with these facts, let us take our study to the oral cavity, and there we will find a culture medium which is kept at the necessary temperature for the growth and development of the micro-organisms. The microscope reveals to us the presence of many species, and when our modern chemistry is more precise we will no doubt discover a more complex mixture of ptomaines as the result of their presence.

If these complex bodies or ptomaines were the only result of these organic growths our oral pathology would be much more simple, but such is not the case. Acids or hydrogen compounds are largely produced as well, and it is these, owing to their energetic effect, that makes for us a much more complicated subject with which to deal.

We are all painfully conscious that the tooth substance is susceptible to the action of acids. Hence, if these facts be admitted, and the organic growth produces either by catalytic action or their excreta a sufficient quantity of acid, which disintegrates tooth substance, it brings us to the practical question of How to arrest and discontinue its action.

To treat a disease does not necessarily require that we must know the cause ; yet, knowing the cause, we can the more scientifically treat the disease.

Therefore, with the subject in hand, it is clear that we need a germicide, an antiseptic and a disinfectant. And it is upon this most important subject that I most earnestly ask your discussion.

We know that all forms of life are the resultant of the process of *nutrition.* Take away the food material and the proper culture medium and we destroy the organic growth. Death of the microbes means one step towards antisepsis.

In the mouth we will find species of microbes that are anærobic ; that is, this form of life will live and flourish in the recesses of tooth-structure without access to oxygen. Hence, simply closing them in with some sort of filling will not stop their development, but they will thrive and produce their acid products to re-act upon the tooth-structure. In the mouth we also find the ærobic microbe which lives by contact with the air.

To say that micro-organisms are the sole cause of dental caries is wrong. They play no part whatever unless there is a fracture or a fissure in a tooth or badly crowded teeth, or some fit place for them to make their home. We can have dental caries as the result of constitutional derangement or some diseased condition of the mucous membrane of the oral cavity and its associate parts. We can have dental caries as the result of the lodgement and decomposition of food material between and around our teeth. Mis-fitting plates, clasps, rough and unfinished fillings will produce local inflammations, which, with their acid products, will cause dental caries.

Therefore, with these facts before us, our mode of treatment will naturally suggest itself, namely, by our modern studies of bacteriology we will seek for an antiseptic or something that will arrest decomposition; we will look for a germicide or some agent that will kill microscopic life; but, knowing that some of these lower forms are very tenacious of life, and that our germicide will kill the adult but will not destroy the spore or egg, if you may so call it, we seek a sporicide, or something that will neutralize the spore or egg, and this we have very effectually in the bichloride of mercury, one to two-thousand parts, and in this we also find the germicide and the antiseptic, so far as microscopic life is concerned. In order that we may guard ourselves against the anærobic microbe, we must use thorough antiseptics in the cavity by means of a solution of bichloride of mercury, one to two-thousand parts, or the application of carbolic acid, or oil of cloves, or absolute alcohol, or all of them as our judgment dictates before we introduce the filling.

If constitutional derangement is the cause, we must direct ourselves to the building up of the patients general health. When the mucous membrane of the oral cavity is diseased in part or whole, we must apply local treatment. When the plate or the clasp or the unfinished filling is the fault, smooth off the corners and cauterize the unhealthy granulations. If decomposed food material packed between and around the teeth is the cause, we must instruct our patients in the most forcible terms that absolute cleanliness is absolutely necessary if they wish to further the use of their dental organs. Give them explicit directions as to *when* and *how* to clean their teeth. See that they are supplied with silk-floss and that they have properly adapted tooth brushes, mouth-wash and tooth powder. See that all deposits upon and around the teeth are thoroughly removed and the enamel of the teeth nicely polished. In fact, thoroughly impress upon their minds that the mouth must be kept in a hygienic condition at all times if they wish to lessen the frequency of decay.

Now, more as a point of discussion, I will mention in connection with this paper the subject of mouth-wash and tooth powders. What compounds or mixtures or solutions shall be prescribed for our patients in order that they may apply oral antiseptics by their own hands? The answer to this question, in order that it may cover all cases, is not easy, but the greatest majority of cases can be covered by prescribing some pleasant and harmless solution, a little quantity of which is to be put into a large quantity of water, with the direction that the mouth and teeth should be thoroughly washed once or twice daily or more times if our judgment so dictates.

If we have any special cases to meet, we can use absolute alcohol, water and glycerine as a vehicle, and then add any germicide or antacid, any anti-phlogistic, any tonic, or any astringent in any quantity or strength as we may deem advisable to meet the requirement of the case. As for a tooth powder the same general rule is to be observed. The more simple the better. Prepared chalk or carbonate of magnesium answer the requirements of the greater number of cases. This base will remove the inspissated mucus and small particles of food that may be clinging to the surfaces of the teeth. To this base, carbonate of magnesium, for special cases we may add any medicinal property that we may think best.

These, then, gentlemen, are in brief my views with regard to the subject of oral antisepsis, and it now remains for the members of this Society to show how far practical and clinical experience confirms or takes issue with the subject in hand.

DISCUSSION.

*Dr. J. W. Wassall:*—The subject which has just been so ably presented is a very important one to dentists. If it were possible to prevent the existence and growth of micro-organisms in the mouth, caries of the teeth could not occur. This, however, would probably not prevent erosion. Prophylaxis depends upon an intelligent appreciation of the pathological conditions to be combatted, and the paper is in this direction. It was Dr. Bogue, of New York, I think, who once said that if it were possible to keep all the surfaces of the teeth absolutely clean, there would be no caries.

*Dr. W. B. Ames:*—I can hardly say more than amen to what Dr. Wassall has just said of the paper and on the subject. Our patients are seldom equipped with proper appliances for cleansing the teeth and surrounding parts, and we are unquestionably doing them a better service in putting them in the way of avoiding, to an extent, the occurrence of decay than in restoring the parts after they have wasted. It is, of course, a question as to what extent caries can be avoided by the use of antiseptic washes, but they are undoubtedly of some benefit, and we ought to be able to give our patients who need it some prescription or the article itself that can be used to advantage. The compounds from spirits glycerine, boracic acid, etc., similar to "Listerine," I have used with good results, and we have hydro-napthol which is efficient in a solution of 1 in 1000 which ought to be a valuable component in a mouth wash.

*Dr. W. H. Jackson:*—How long will your antiseptics remain during the night with the flow that there is of saliva? Cleanliness is all very good, but you must bring the system to such a condition that the micro-organisms will not develop, that the system will not be subject to them. Our greatest difficulty is to fight the general diseases of the system. Hence we cannot hope to have any "cure all" until we place the system in such a condition that the micro-organisms will not germinate.

*Dr. W. B. Ames:*—Where do the germs come from?

*Dr. Jackson:*—From the earth, the air, our food, etc. If we could breathe through a tube containing sulphuric acid, we could kill them, and thus prevent their getting at the teeth.

*Dr. Ames:*—Then the mouth-breather is more apt to have diseased teeth than he who breathes naturally.

*Dr. Jackson:*—Undoubtedly. In the treatment of certain certain diseases in the mouth, you will find acetic acid a very good thing, also alcohol.

